# Long Noncoding RNA MEG3 Interacts with p53 Protein and Regulates Partial p53 Target Genes in Hepatoma Cells

**DOI:** 10.1371/journal.pone.0139790

**Published:** 2015-10-07

**Authors:** Juanjuan Zhu, Shanshan Liu, Fuqiang Ye, Yuan Shen, Yi Tie, Jie Zhu, Lixin Wei, Yinghua Jin, Hanjiang Fu, Yongge Wu, Xiaofei Zheng

**Affiliations:** 1 Beijing Institute of Radiation Medicine, Beijing, China; 2 College of Life Sciences, Jilin University, Changchun, China; 3 School of Life Science and Technology, China Pharmaceutical University, Nanjing, China; 4 Department of Pathology, General Hospital of PLA, Beijing, China; German Cancer Research Center, GERMANY

## Abstract

Maternally Expressed Gene 3 (*MEG3)* encodes a lncRNA which is suggested to function as a tumor suppressor. Previous studies suggested that MEG3 functioned through activation of p53, however, the functional properties of MEG3 remain obscure and their relevance to human diseases is under continuous investigation. Here, we try to illuminate the relationship of MEG3 and p53, and the consequence in hepatoma cells. We find that transfection of expression construct of MEG3 enhances stability and transcriptional activity of p53. Deletion analysis of MEG3 confirms that full length and intact structure of MEG3 are critical for it to activate p53-mediated transactivation. Interestingly, our results demonstrate for the first time that MEG3 can interact with p53 DNA binding domain and various p53 target genes are deregulated after overexpression of MEG3 in hepatoma cells. Furthermore, results of qRT-PCR have shown that MEG3 RNA is lost or reduced in the majority of HCC samples compared with adjacent non-tumorous samples. Ectopic expression of MEG3 in hepatoma cells significantly inhibits proliferation and induces apoptosis. In conclusion, our data demonstrates that MEG3 functions as a tumor suppressor in hepatoma cells through interacting with p53 protein to activate p53-mediated transcriptional activity and influence the expression of partial p53 target genes.

## Introduction

ncRNAs are functional RNA molecules that are not translated into proteins, including transfer RNAs (tRNAs), ribosomal RNAs (rRNAs), microRNAs (miRNAs), small nucleolar RNAs (snoRNAs), Piwi-RNAs and long non-coding RNAs (lncRNAs) [1]. A growing number of literatures point to the critical regulatory role of ncRNAs in normal cellular physiological processes as well as the contribution of aberrant ncRNA expression to cancer biology [2]. Among them, lncRNAs which are widely distributed in mammals, are a class of RNA molecules with more than 200 nucleotides that function as RNAs with little or no protein-coding capacity [3]. Recently, researches demonstrated that lncRNAs played an important role in cell physiological activities, and participated in development of varieties of tumors and other diseases via multiple levels as epigenetics, transcription and post-transcription [4–7]. Deregulation of lncRNAs has been proposed to be involved in hepatocarcinogenesis, such as HEIH, HUCL, MVIH, H19, TUC338 and MEG3 [8–13].

*MEG3* is homologous with mouse maternally imprinted gene *Gtl2*, a maternally expressed imprinted gene first identified by Miyoshi N *et al*. in 2000 [14]. *MEG3* belongs to *DLK1-MEG3* imprinted region at chromosome 14q32.3 in human and transcripts an mRNA-like RNA with a length of ~1.6kb [15]. Due to the TATA box within the gene promoter and RNA transcript with a poly(A) tail, MEG3 is a target gene of RNA polymeraseⅡ [16]. Sixteen different variants generated via alternative splicing of 10 exons within *MEG3* gene have been proven, as sixteen human *MEG*3 cDNA isoforms have been reported. Among them, variant 1 (NR_002766), otherwise known as MEG3, is the predominant transcript [17, 18].

Previous reports demonstrated that MEG3 is highly expressed in normal human tissue. However, its expression is lost or decreases in major human tumors, such as meningioma, colon cancer, nasopharyngeal carcinoma, and leukemia [19]. Moreover, it has been reported that MEG3 functions as a tumor suppressor through activating p53 pathway [19–22]. P53 functions as a transcriptional factor through mediating tumor suppression. Previous data demonstrated that MEG3 is capable of inducing a significant increase in p53 protein level. However, the function of *MEG3* gene has not yet been completely understood, especially, little is known about how lncRNA MEG3 plays a regulatory role collaborated with p53. Here, we uncover a functional mechanism of MEG3 regulation via interacting with p53 protein to influence p53 target genes, and executing its tumor suppressor function.

## Materials and Methods

### Patients and Tumor samples

23 pairs of HCC samples and adjacent non-tumor tissues were obtained from surgical specimens at General Hospital of the People’s Liberation Army (Beijing, China) after informed consent. These samples used in this study have been described in previous publication [23]. Clear hepatocellular carcinoma was diagnosed histopathologically. All these specimens were snap-frozen in liquid nitrogen after excision. Clinical characteristics of the patients included in this study were in Table A in S1 File. This study was approved by the Ethics Committee of General Hospital of the People’s Liberation Army. Informed consent was obtained from each participant and the methods were carried out in accordance with the approved guidelines.

### Cell lines, Cultures and Luciferase reporter assay

HepG2, SK-Hep–1, HEK293, HCT116 cell lines were purchased by ATCC (Manassas, VA) and cultured in Dulbecco’s modified Eagle medium (DMEM) (GIBCOBRL, Grand Island, NY) supplemented with 10% fetal bovine serum, 100 U/mL penicillin and 100 μg/mL streptomycin in a humidified incubator at 37°C with 5% CO_2_. For luciferase assay, adherent cells in 24-well plates were cotransfected with 100 ng firefly luciferase reporter vector named pG13L (p53-responsive reporter plasmid, containing 13 repeats of p53-responsive element), 1 ng of control vector containing Renilla luciferase named pRL-TK and 300 ng pcDNA3.0-MEG3 or pcDNA3.0 using Lipfectamine 2000 (Invitrogen, Carlsbad, CA) as indicated. Luciferase activities were measured using dual luciferase assays (Promega, Madison, WI) 48h after transfection according to manufacturer’s instruction.

### Plasmid construction and Construction of hepatoma cell lines stably expressing MEG3

For the expression of MEG3, full-length Homo sapiens MEG3 sequences (NR_002766) were cloned into *Kpn*Ⅰ and *Xho*Ⅰ site of the pcDNA3.0 vector. pG13L vector was a gift from Professor Lingqiang Zhang of Beijing Institute of Radiation Medicine.

To obtain cell line stably expressing MEG3, the expression vector pcDNA3.0-MEG3 was transfected into Hep-SK–1 cells and screened with G418 (1000 μg/ml). QRT-PCR was performed to examine the high expression of MEG3 at the RNA level. The cell line with stably expressing empty vector pcDNA3.0 served as control.

### RNA extraction and Quantitative Reverse Transcription PCR

Total RNA from cells and 23 pairs of tissues were extracted using TRI regents (Sigma-Aldrich, St. Louis, MO) according to the manufacturer’s protocol. Reverse transcription was performed using 1 μg reverse transcription primes Odigo(dT) and 2μg total RNA in ImProm-II^TM^ Reverse Transcription System (Promega). Quantitative real-time PCR (qRT-PCR) was performed using SYBR Premix EX Taq^TM^ (TaKaRa, Dalian, China). Primers of MEG3 and other genes as well as glyceraldehydes-3-phosphate dehydrogenase (GAPDH) as control were presented in Table B in S1 File.

### Growth suppression assay

Growth suppression of hepatoma carcinoma cell line HepG2 by MEG3 was measured by cell proliferation assay and colony formation assay. The viability of cells in post-transfection was determined using Cell Counting Kit–8 (Dojindo, Japan). For colony formation assay, cells were harvested 24 hours post transfection and 2×10^3^ cells were seeded in 6-well culture plates in DMEM containing 10% FBS and 1000 μg/ml neomycin, and incubated for 1 week for proper colony formation. Thereafter, culture media was discarded and the cells were washed two times with cold PBS. Then, the cells were fixed by treatment with 95% ethanol for 15 minutes and stained with 0.1% crystal violet solution for 15 minutes.

### Flow-cytometric analysis of apoptosis

HepG2 cells transfected with pcDNA3.0 or pcDNA3.0-MEG3 after serum-starved for 24 hours were harvested by trypsinization. Following double staining with FITC-Annexin Ⅴ and Propidium isodide (PI), the cells were analyzed using flow cytometry (FACScan, BD, Biosciences). Cells were discriminated into four parts including viable cells, dead cells, early apoptotic cells, and apoptotic cells. This experiment was independently performed at least three times.

### RNA pulldown assay

Biotin-labeled lncRNA-MEG3 were *in vitro* transcribed with a Biotin RNA Labeling Mix (Roche, Indianapolis, IN) and the T7 RNA polymerase (Promega), be treated with RNase-free DNase I (Promega) and purified with MEGAclear^TM^ Kit (Ambion, Austin, USA). Five micrograms of biotinylated MEG3 RNA was heated to 70°C for 5 minutes, put on ice for 2 minutes, then supplied with RNA structure buffer (10 mM MgCl_2_, 10 mM Tris pH 7, 0.1 M KCl) and then shifted to room temperature (RT) for 20 minutes to allow proper secondary structure formation. 10^7^ HEK293 cell pellets were re-suspended in 1ml lysis buffer (150 mMKCl, 25 mMTris pH 7.4, 0.5 mM DTT, 0.5% NP40, 1 mM PMSF, RNase inhibitor (TaKaRa) and protease inhibitor (Roche Complete Protease Inhibitor Cocktail Tablets). Debris was pelleted after centrifugation at 12,000 RPM for 15min. 10μl of the lyses was transferred to a microfuge tube as 1% input sample. Folded RNA was mixed with 1ml lyses of 10^7^ cells extract in lysis buffer and incubated at RT for one hour. 20μl washed High Capacity Streptavidin Agarose Beads (Thermo, Rockford, IL) were added to the binding reaction and incubated for one hour at RT. Beads were washed two times in low salt wash buffer for 5 minutes with rotation, and two times in high salt wash buffer. Then, the beads were boiled in SDS buffer, and the associated protein was detected by western blot.

Recombinant GST-P53 was purified from BL21 bacterial using GST-agarose affinity chromatography. 2 μg of biotinylated RNA was incubated with 5μg of GST-P53 protein in 200μl binding buffer (50 mM TrisCl 7.9, 10% Glycerol, 100 mMKCl, 5 mM MgCl_2_, 10 mM β-ME, 0.1% NP–40) for one hour at RT. 20 μl washed High Capacity Streptavidin Agarose beads (Thermo) were added to each binding reaction and incubated at RT for 30 minutes, and then beads were washed two times in low salt wash buffer for 5 minutes with rotation, and two times in high salt wash buffer. Retrieved protein was detected by standard western blot technique.

### RNA immunoprecipitation

10^7^ cells were washed twice in PBS with PMSF and pelleted. The pellet was lysed in 1 ml of RIPA buffer (50 mM Tris, pH 7.4, 150 mM NaCl, 1 mM EDTA, 0.1% SDS, 1% NP–40, and 0.5% sodium deoxycholate, 0.5 mM DTT and 1 mM PMSF/cocktail), incubated at 4°C for 30 minutes with rotation. The lysate was obtained after centrifugation at 13,000 RPM for 15 minutes at 4°C. Two parts of 10 μl of the supernatant of lysate were removed as 1% input. One part was used to test the expression of RNA-binding protein of interest by western blotting. The other part was utilized to retrieve RNA for comparison in RT-PCR. 5~10μg antibody and equal negative control IgG of the same species were added and binding samples were incubated for at 4°C for 4 hours with rotation. Samples were washed two times in low salt wash buffer (RIPA buffer), two times in high salt wash buffer (50 mMTris, pH 7.4, 1 M NaCl, 1 mM EDTA, 0.1% SDS, 1% NP–40, and 0.5% sodium deoxycholate). 100 μl each out of 1ml of the beads suspension during the last wash was removed to test the efficiency of immunoprecipitation by western blotting. Remained beads and 1% input sample were treated with proteinase K buffer containing 117 μl of RIP wash buffer, 15 μl of 10% SDS, 18 μl of 10 mg/mL proteinase K at 55°C for 30 minutes with shaking to digest the protein. RNA samples were extracted with phenol and chloroform. Co-precipitated RNAs were detected by qRT-PCR.

### Western Blot

Western blot analysis was performed as described previously [24]. Briefly, cells were lysed in RIPA buffer supplemented with protease inhibitor cocktail (Roche) for 30 min on ice to extract total protein. Protein concentration was determined by BCA protein assay (Pierce, Rockford, IL). The protein samples were boiled in 1× sodium dodecyl sulfate buffer for 10 minutes. Protein with the same amount was subjected to sodium dodecyl sulfate-polyacrylamide gel electrophoresis and transferred onto poly-vinylidene fluoride membranes. The membranes were blocked with 5% skim milk, and probed with the antibody. It was visualized by enhanced chemiluminescence reagents super signal (Pierce). Mouse monoclonal anti-p53 antibody (sc–126; Santa Cruz, Santa Cruz Biotechnology, CA), mouse monoclonal anti-β-actin antibody (Proteintech, Proteintech group, USA), anti-GST antibody (MBL, Medical & Biological Laboratories Co., Japan), mouse monoclonal anti-FLAG antibody (MBL) were used for western blotting.

### Microarray analysis and computational analysis

Sample preparation and microarray hybridization were performed by Emei Tongde Bio-tech, Beijing P.R. China. The Illumina TotalPrep RNA Amplification Kit was used to for generating biotinylated, amplified RNA for hybridization with Human HT–12 V4.0 Expression Beadchip. After having washed the slides, the arrays were scanned using Illumina BeadChip Reader and data analyzed using Illumina BeadStudio software. Average was carriey for sample normalization. Sample intensities are scaled by a factor equal to the ratio of average intensity of virtual sample to the average intensity of the given sample. Background is subtracted prior to the scaling. Differentially expressed genes were identified through fold change filtering (Fold Change ≥ 2.0 or ≤ 0.5) and paired t-test (p < 0.01). The microarray data discussed in this paper has been deposited in NCBI Gene Expression Omnibus and the GEO accession number is GSE69916. Compared with 287 p53 target genes whose data come from transcriptional regulatory element database (TRED), we found 34 of these p53 target genes showed consequent changes in gene expression in stable cell line overexpressing MEG3 (Tables 1 and 2).

**Table 1:** Up-regulated p53 target gene in MEG3 microarray.

Gene Symbol	Log2 ratio(MEG3/pcDNA3.0)	Detection Pval
**LRIG1**	4.553059398	0
**IL6**	3.861707287	0
**EGR1**	3.134976596	0
**DDIT4**	3.035782246	0
**IL8**	2.832647013	0
**EEF1A2**	2.633989115	0.0013
**BTG1**	2.613107268	0
**ABCC3**	2.55841459	0.00002
**GDF15**	2.141727055	0
**CEBPA**	2.106515831	0.0026
**TGFA**	2.027331015	0
**SESN2**	1.946581156	0
**TP53INP1**	1.791814071	0
**TP53I11**	1.631709645	0.00729
**PRKAB1**	1.511265613	0
**GADD45A**	1.440580184	0
**IER3**	1.243608155	0
**BIRC3**	1.209765266	0
**IFI16**	1.190804468	0
**BIRC2**	1.16998912	0
**RRM2B**	1.144503259	0

**Table 2:** Down-regulated p53 target gene in MEG3 microarray.

Gene Symbol	Log2 ratio(MEG3/pcDNA3.0)	Detection Pval
**THBS1**	-1.122491311	0
**TK1**	-1.407363571	0
**CDC25C**	-1.629947344	0.00002
**CCNB1**	-1.632181656	0
**LIG1**	-1.768076127	0.0013
**TYMS**	-1.843215092	0
**EZH2**	-1.90671027	0
**MCM7**	-2.025617534	0.00206
**POLD1**	-2.149440579	0.0013
**BIRC5**	-2.173319325	0
**MCM2**	-2.178534676	0.0013
**PLK1**	-2.677610317	0.0013
**E2F2**	-3.54962012	0.0013

### Statistical analyses

All experiments were independently performed at least three times. The values are presented as mean ± SD. Differences were assessed by two-tailed Student’s *t*-test. Analysis was used Microsoft Excel 2008 software and GraphPad prism (prism 5 for windows). *P*<0.05 was considered statistically significantly.

## Results

### MEG3 enhances stability and transcriptional activity of p53

**Fig 1:**
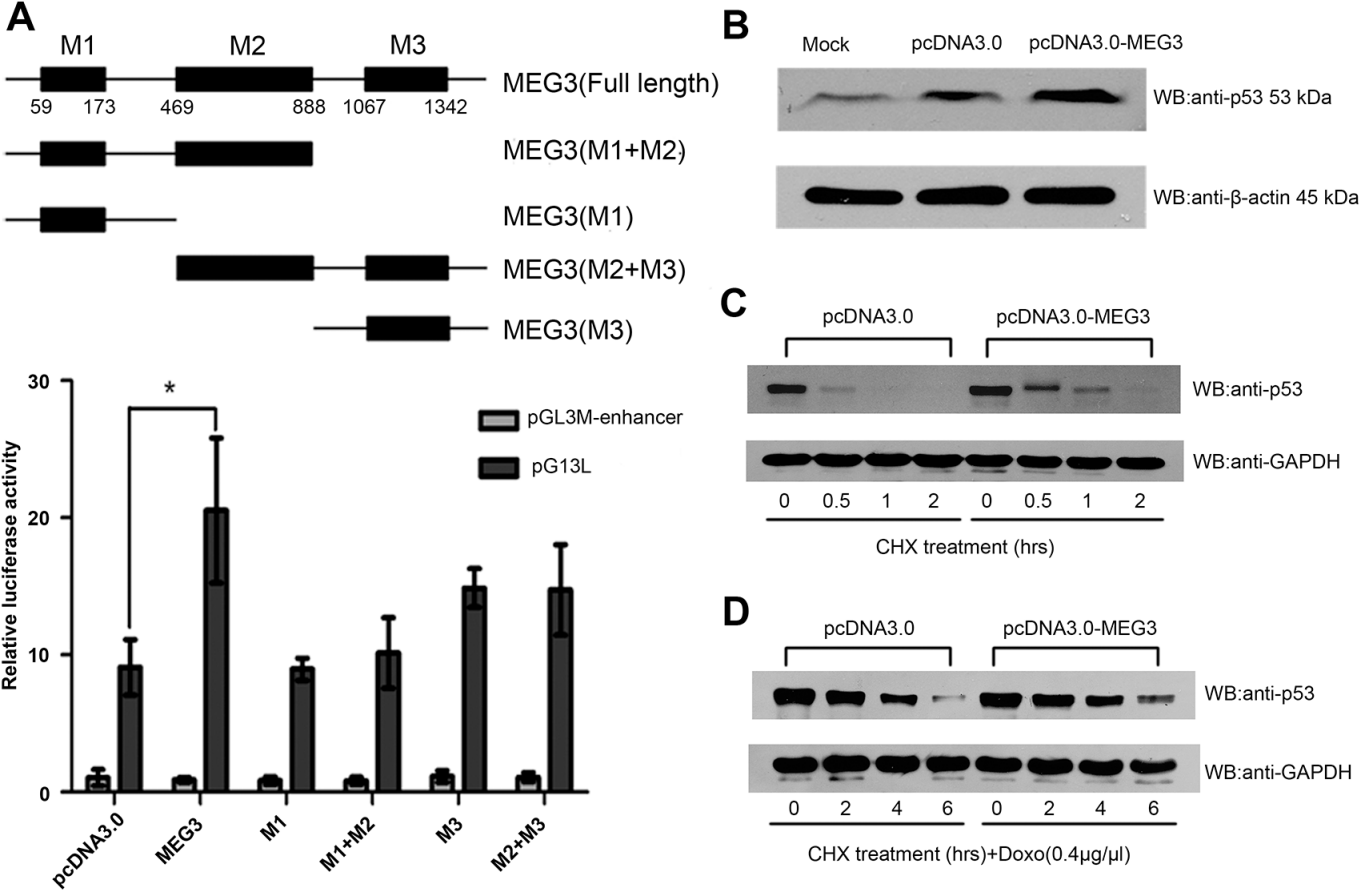
MEG3 enhanced stability and transcriptional activity of p53. (A) Reporter assays detected stimulation of p53-mediatd transactivation by MEG3 deletion mutants M1, M1+M2, M3, M2+M3 in HepG2 cells. The value are means of three independent experiments ±S.D, * P<0.05. (B) Cropped blots show the increased level of p53 protein 48h after transfection of the pcDNA-MEG3 in HepG2 cells. (C) Analysis of the effect of MEG3 overexpression on the half-life of p53. Cropped blots show the relative abundance of p53 after treated with translation inhibitor CHX for various amount of time in HepG2 cells (0, 0.5,1, 2h) overexpressing MEG3. (D) Analysis of the effect of MEG3 overexpression on the half-life of p53. Cropped blots show the relative abundance of p53 after treated with translation inhibitor CHX for various amount of time in doxo-induced HepG2 cells (0, 2,4, 6h)overexpressing MEG3.

Previous studies suggested that MEG3 functioned as a tumor suppressor through activation of p53, leading to increase in p53 protein levels and stimulate p53-dependent transcription in colon and brain cancer cells [22]. To confirm the possibility that p53 may be involved in anti-proliferation function of MEG3 in hepatoma cells, we co-transfected a p53-responsive reporter plasmid (pG13L) with MEG3 expression vector into HepG2 cells. Results of luciferase assay revealed that MEG3 could enhance the transcriptional activity of p53 (Fig 1A). MEG3 contains three conserved motifs, designated as M1, M2 and M3 [16]. To validate the functional importance of these motifs, we generated a series of MEG3 deletion mutants. Each mutant was tested for its ability to activate p53-mediated transcriptional activity in luciferase reporter assay. As shown in Fig 1A, compared with full length of MEG3, each deletion mutants failed to stimulate the transcriptional activity of p53 transactivation in HepG2 cells, indicating that full length of MEG3 and intact structure are critical to activate the transcriptional activity of p53. Meanwhile, we also found that overexpression of MEG3 lead to an increased level of p53 protein in HepG2 (Fig 1B), but had no effect on the p53 transcript (data was not shown). Based on this observation, we speculated that enforced expression of MEG3 might affect the stability of the p53 protein. Then, we directly measured the stability of p53 protein under conditions where MEG3 was overexpressed by treating HepG2 cells and doxorubicin induced-HepG2 cells with cycloheximide in different time points and noted that overexpressed of MEG3 significantly stabilized p53 expression (Fig 1C and 1D).

### MEG3 interacts with p53 protein directly

**Fig 2:**
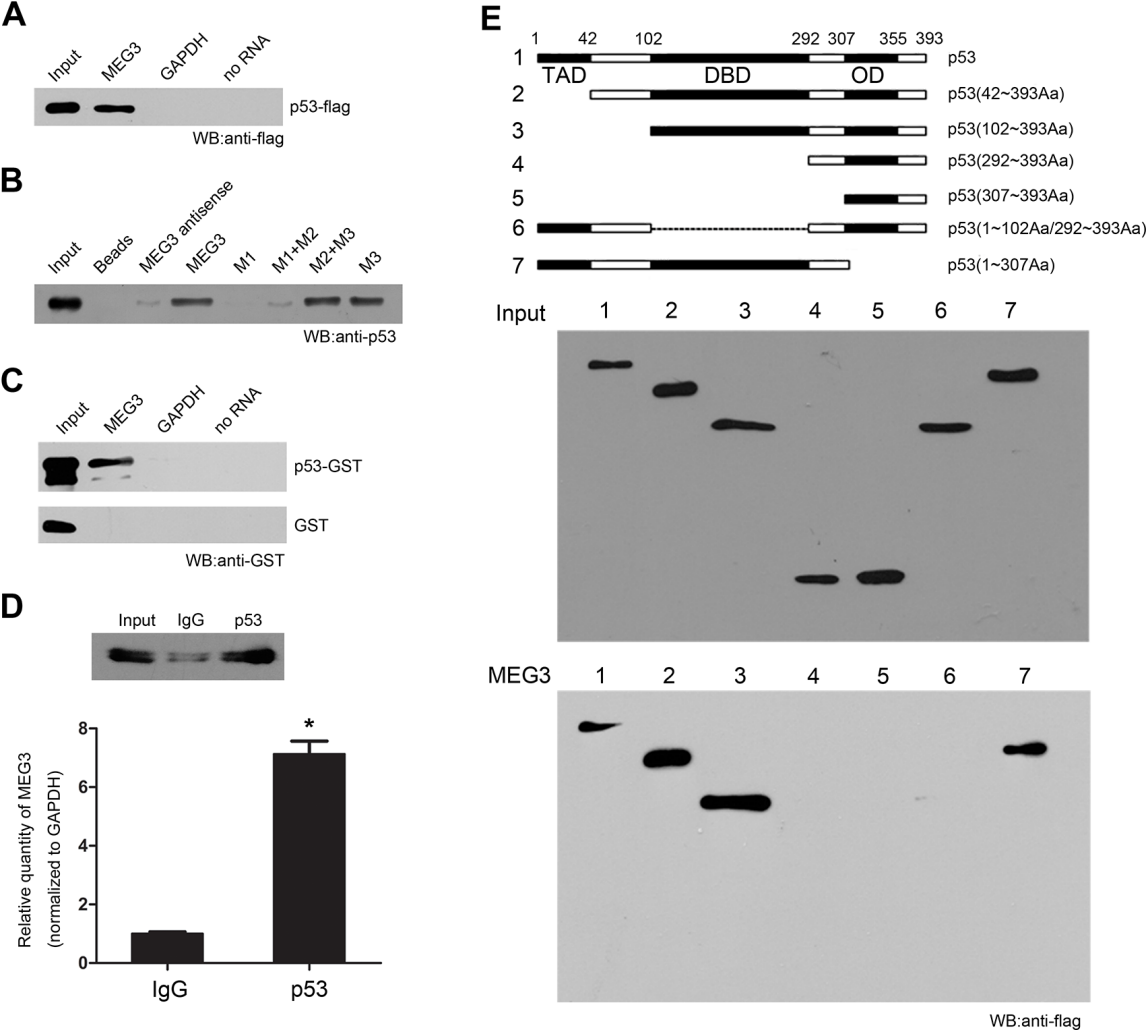
MEG3 interacted with p53 protein directly. (A) Western blot of p53 protein bound to in vitro transcribed biotinylated MEG3 incubated with HEK293 cell extract transfected with Flag-p53 vector. (B) Western blot of p53 protein bound to in vitro transcribed biotinylated MEG3 and deletion mutations RNA incubated with doxo-induced HepG2 cell extract (MEG3 deletion mutants M1, M1+M2, M3, M2+M3 are shown in Fig 1A). (C) In vitro transcribed biotinylated MEG3 retrieved purified GST-p53 but not GST. (D) RIP experiments were performed using an antibody against the p53 on extracts from doxo-induced HepG2 cells. The purified RNA was used for qRT-PCR, and the enrichment of the lncRNA MEG3 was normalized to GAPDH (upper, western blot of p53 protein after immunoprecipitation). Data was relative to mock-IP (IgG). (E) A series of p53 deletion mutants which were flag-tagged was treated as in (A), and association was detected by anti-Flag. Up represents successful expression of p53 deletion mutants. Down represents in vitro transcribed biotinylated MEG3 RNA was incubated with HEK293 cell extract which was transfected with p53 deletion mutants and associated proteins were detected by anti-Flag.

Our results indicated that MEG3 could enhance stability and transcriptional activity of p53 in hepatoma cells. One important way that lncRNAs play their functions is to interact with certain critical proteins, which imply that MEG3 might affect p53 transcriptional activity and target gene expression via interacting with p53 protein. Therefore, RNA pulldown was performed to identify whether p53 protein was associated with lncRNA MEG3. As was shown in Fig 2A and 2B, purified biotinylated MEG3 RNA specifically retrieved p53 from HEK293 cell extract which was transfected with Flag-p53 expressing vector and from doxo-induced HepG2 cells extract. Further, biotinylated MEG3 bound to purified GST-p53 but not GST *in vitro* (Fig 2C). Results of RIP assay revealed that antibody against p53 could result in higher enrichment of MEG3 RNA in doxo-induced HepG2 cells, compared with the IgG control (Fig 2D). To further identify which domain of p53 is necessary for interacting with lncRNA MEG3, a series of vectors expressing p53 deletion mutants were constructed and RNA pulldown experiment was performed. The result showed that DBD of p53 was responsible for the direct association with MEG3 (Fig 2E). Moreover, in order to identify which domain of MEG3 is critical for the interaction, we used MEG3 deletion mutants to bind p53 and found that M3 domain is important for the interaction (Fig 2B). In summary, results of *in vivo* RIP and *in vitro* RNA pulldown demonstrated the direct interaction between p53 and MEG3.

### MEG3 regulates the expression of partial p53 target genes

**Fig 3:**
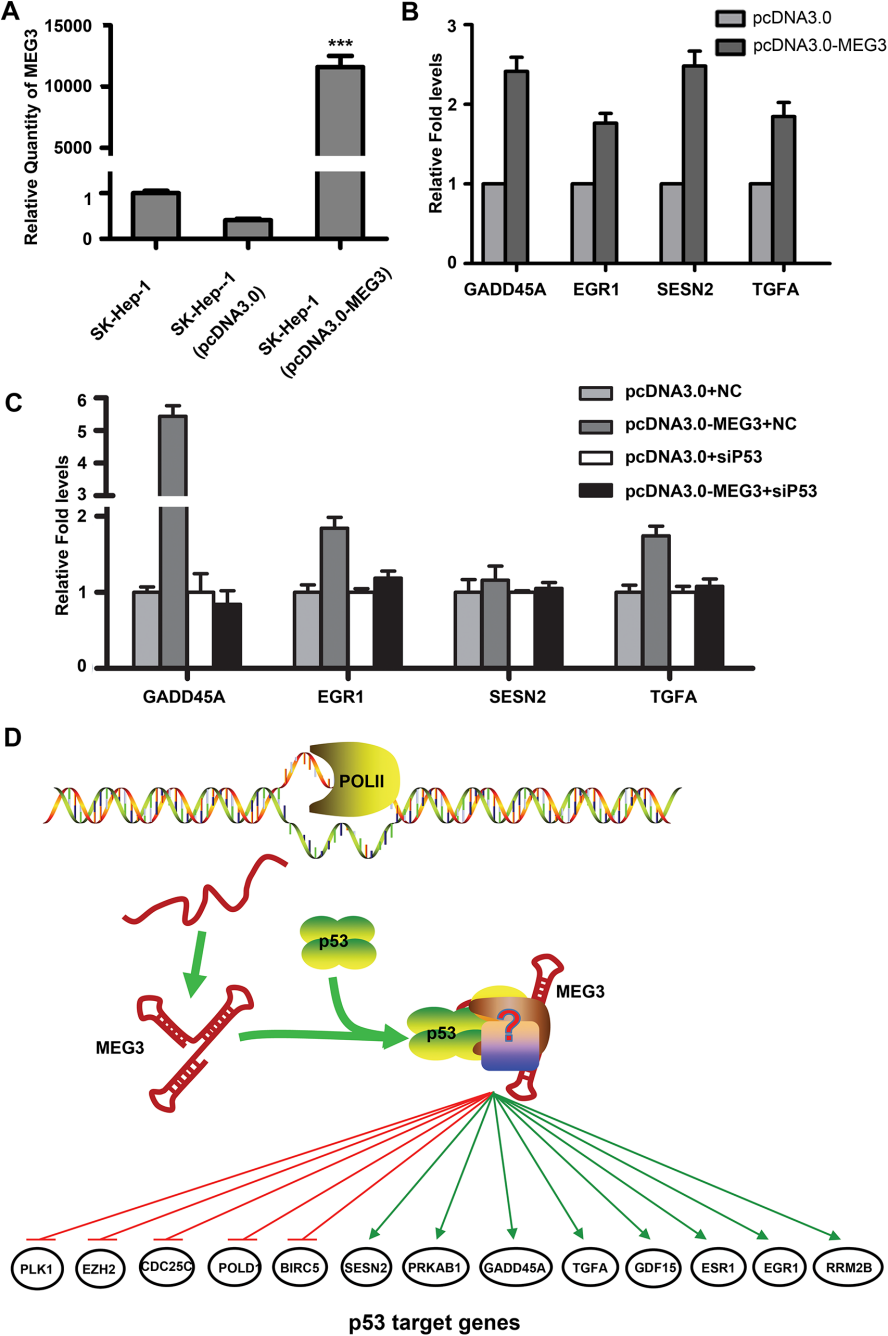
MEG3 regulated part of p53 target genes. (A) SK-Hep–1 hepatocellular carcinoma cell lines with stably expression of MEG3 were determined by qRT-PCR. The value are means of three independent experiments ±SD, *** P<0.001. (B) Expression of GADD45A, EGR1, SESN2 and TGFA was examined by qRT-PCR and normalized to GAPDH. The bars represent the relative fold change of these genes in SK-Hep–1 after transfection with pcDNA3.0-MEG3 compared with blank vector pcDNA3.0. (C) Expression of GADD45A, EGR1, SESN2 and TGFA was examined by qRT-PCR and normalized to GAPDH after knockdown p53 level in HepG2 cells. The bars represent the relative fold change of these genes in HepG2 cells after co-transfection of pcDNA3.0 and NC, pcDNA3.0-MEG3 and NC, pcDNA3.0-MEG3 and sip53, pcDNA3.0 and sip53, respectively. (D) A schematic diagram illustrating how MEG3 can function as tumor suppressor through interactions with p53.

The most significant function of p53 is to act as a transcription factor that directly or indirectly regulates many target genes. To quantify further analysis of the functional mechanism of MEG3, we constructed SK-Hep–1 hepatoma cell lines that stably over-expressed MEG3 (Fig 3A). In order to investigate which p53 target genes were affected by MEG3, we compared the differential genes between stable SK-Hep–1 cell line overexpressed MEG3 and control SK-Hep–1 cell line transfected with pcDNA3.0 by mRNA expression microarray analysis. Through bioinformatics analysis of microarray data, compared with 287 p53 target genes whose data come from transcriptional regulatory element database (TRED) [25, 26], we found 34 of these p53 target genes showed consequent changes in gene expression in stable cell line overexpressing MEG3 (Tables 1 and 2) including 21 up-regulated genes which principally involved in negative regulation of apoptosis, cell death, cell cycle and cell proliferation and 13 down-regulated genes which mainly participated in positive regulation of cell cycle and DNA replication. For example, GADD45A which is a p53-responsive stress protein leads to growth arrest and functions as tumor- and autoimmune suppressors [27]; CDC25C phosphatase which triggers cellular entry into mitosis is a target for transcriptional downregulation by p53 and this repression can be shown to contribute to p53-dependent growth arrest [28]. Using qRT-PCR, we validated the changes in the cellular levels of part of p53 target genes (Fig 3B and Figure A in S1 File.). The results showed that GADD45A, EGR1, SESN2 and TGFA were up-regulated in SK-Hep–1 after overexpression of MEG3, which is consistent with the microarray data. Moreover, to further identify whether the regulations of these genes by MEG3 depended on p53, we used siRNA-p53 to knock down p53 level and validate the changes of these genes by RT-qPCR (Fig 3C). The results showed that MEG3 overexpression could not regulate genes level after knockdown p53. All these data suggest that MEG3 not only affects p53 stability but regulates, at least in part, the transactivation of selected p53 target genes.

### MEG3 is down-regulated in HCC tissues and inhibits the growth of hepatoma cells

**Fig 4:**
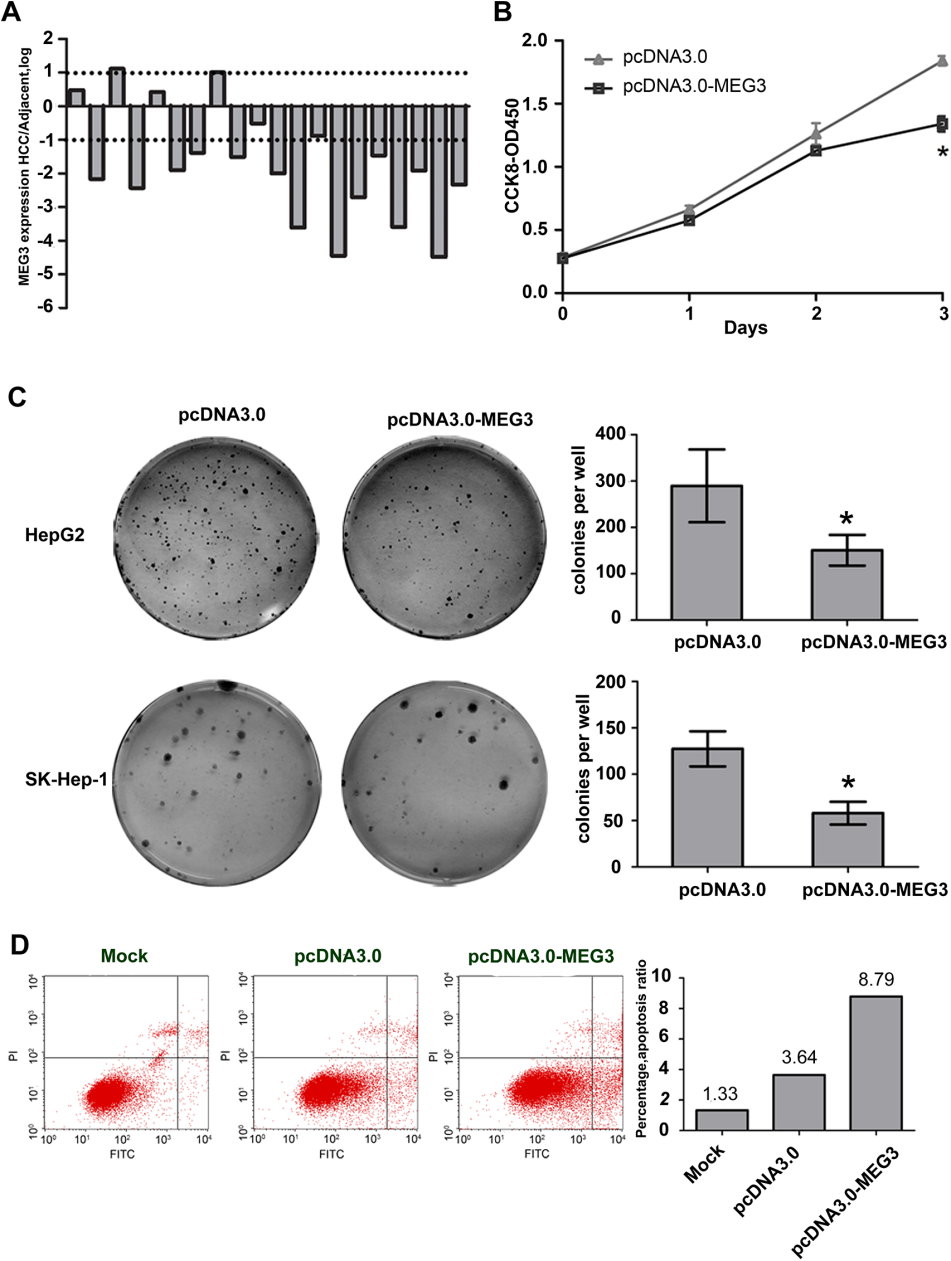
Overexpression of MEG3 inhibited growth of hepatoma cells and induces cell apoptosis. (A) MEG3 was remarkably reduced in HCC tissues. Reduced and undetected samples account for 82% and 13% of the total samples. Total RNA was extracted from human HCC and adjacent nontumorous tissues with Trizol. Expression of lncRNA MEG3 was examined by qRT-PCR and normalized to GAPDH. The bars represent the ratio of MEG3 between HCC and adjacent nontumorous tissues (log scale). (B) The effects of MEG3 on viability of cells were detected by using Cell Counting Kit–8. The value are means of three independent experiments ±SD, * P<0.05. (C) The effects of MEG3 on cell growth were detected by using colony formation assay in HepG2 and SK-Hep–1. The value are means of three independent experiments ±SD, * P<0.05. (D) Ectopic expression of MEG3 induced cell apoptosis. Cell apoptosis distribution of HepG2 cells transfected with pcDNA3.0 or pcDNA3.0-MEG3 after serum-starved for 24 hours. The apoptotic rates of cells were detected by flow cytometry. This experiment was independently performed at least three times and the change tendency is the same. One of the results was shown in Figure.

To identify MEG3 that is altered in expression in HCC, we detected the expression levels of MEG3 in 23 pairs of HCC tissues and adjacent non-tumor tissues. The results showed that MEG3 were remarkably reduced twofold or more in 14 HCC samples compared with the adjacent non-tumoral samples, and not detected in the other 3 HCC samples (Fig 4A). To assess the functional consequences of deregulated MEG3 expression, we transfected hepatoma cells with MEG3 expressing vector (pcDNA3.0-MEG3), and detected its ability to inhibit cell growth by cell-counting kit–8 assay. The results indicated that overexpression of the MEG3 in HepG2 cells resulted in a decrease in cell growth rate compared with the control vector (Fig 4B). Moreover, enforced expression of MEG3 resulted in suppression of colony formation in SK-Hep–1 and HepG2 (Fig 4C). Taken together, these results demonstrated that MEG3 can inhibit the proliferation of human hepatoma cells. Next, to determine why MEG3 can inhibit hepatoma cell growth, cell cycle and apoptosis were analyzed by flow cytometry. The results showed that there was no significant change in cell cycle distribution of HepG2 cells transfected with MEG3 compared with control vector (data was not shown). The analysis of apoptosis indicated that ectopic expression of MEG3 could enhance serum starvation induced apoptosis in HepG2 cells (Fig 4D).

## Discussion

In this study, we have identified that ectopic expression of *MEG3* gene in human tumor cells significantly inhibits proliferation and induces apoptosis. Moreover, overexpression of MEG3 resulted in increasing of p53 protein and activated its transcription activity. Furthermore, we found that MEG3 could interact with DBD of p53, and play regulatory roles.

Normally, half-life of p53 is short according to the degradation mediated by ubiquitin complex [29]. We observed that overexpression of MEG3 significantly stabilized p53 expression which leads to an increased level of p53 protein. Therefore, it is likely that MEG3 activates p53 through inhibition of p53 ubiquitination and blockage of p53 degradation. Y zhou *et al*. have found that MDM2 expression was repressed by MEG3, suggesting that down-regulation in MDM2 protein levels contributes to p53 activation by MEG3 [22]. Meanwhile, there is other possible mechanism by which MEG3 activates p53. A dizzying number of cofactors involved in the pathway of p53 network coordinate with p53. Indeed, as the most in-depth transcription factor, p53 tetramer associated with multiple regulators binds to its response elements where it could recruit myriad of transcriptional co-regulators such as chromatin remodeling complexes, histone modifying enzymes, and components of transcription machinery to modulate activity of RNA polymerase at target loci [30]. So it is possible that MEG3 which can be folded into a complicated structure can act as co-activator of p53 to activate its transcriptional activity. While p53 is active as a homotetramer whose tetramerization is pivotal for its function and plays a vital role in the regulation of p53 activity [31], it is possible that MEG3 contributes to the tetramization of p53 leading to activation of p53.

The results of RIP, RNA pulldown and deletion mapping demonstrated the direct interaction between MEG3 and DNA binding domain of p53 is responsible for the direct association of p53 with MEG3. The architecture of p53 protein contains three major domains: N-terminal transactivation domain (TAD), central sequence-specific DNA binding domain (DBD), and C-terminal regulator region containing oligomerization domain (OD)[32]. Disruption of p53 pathway occurring in a variety of cancers correlated with the tumorigenic mutants that are defective in DNA binding and consequently lose the function of transcriptional activation [33]. Therefore, it is clear that DBD is essential for the activity of p53 to bind specific response element. According to our result that DBD of p53 is responsible for the direct association with MEG3, it would lead to inhibition of p53, however, we observed that MEG3 enhance transcriptional activity of p53. Thus, we speculated that this is a dynamic process in cells that the compound which is formed by association of p53 and MEG3 was recruited to the specific target gene, then MEG3 dissociated and p53 started to activate target gene.

In this article, it is intriguing that although p53 protein level was increased after ectopic expression of MEG3, results showed that part of p53 target genes were varied, such as GADD45A, EGR1, SESN2 and TGFA. The p53 transcriptional program is regulated in a stimulus-specific fashion [34, 35], whereby distinct subset of p53 target genes are induced in response to different stimulation [30]. Therefore, it is possible that interaction between MEG3 and p53 alters p53 binding propensity which may be due to the primary, secondary or senior structure of MEG3. GADD45A, a p53-regulated and DNA damage responsive gene, plays an important role in suppressing cell proliferation, mediating cell cycle arrest, promoting apoptosis, inducing DNA repair, and stabilizing genomics [36]. TGFA, a direct target gene of p53, encodes a growth factor that is a ligand for the epidermal growth factor receptor, which activates a signaling pathway for cell proliferation, differentiation and development [37]. These genes have been associated with many types of cancers. Tumor suppression of MEG3 may be implemented by the function of these target genes of p53.

P53 is a crucial tumor suppressor which is involved in preventing cancer through acting as a transcription factor to regulate its target genes [38–40]. In this article, we first found that lncRNA MEG3 could interact with p53 protein to activate p53 target genes. Association between a transcription factor and lncRNA is not yet the first discovery, as previous studies have found that lncRNA PANDA could interact with the transcription factor NF-YA to limit expression of pro-apoptotic genes under DNA damage [41]. Though there were many studies on the regulatory mechanism of p53 function before, lncRNA involved in the regulation of p53 function is a wholly novel field. How to build the relationship among lncRNA, protein and chromatin remains to be studied. Our data proposed a hypothesis that the association of p53 and MEG3 formed a compound to enhance stability of p53, and the compound was recruited to the specific target gene according to the selectivity of MEG3 sequence or structure, then MEG3 dissociated and p53 started to activate target gene to play a tumor suppressor role (Fig 3D).

## Supporting Information

S1 File Figure A- Deregulated p53 target genes in stable SK-Hep–1 cell line overexpressed MEG3. Expression of p53 target genes was examined by qRT-PCR and normalized to GAPDH. The bars represent the relative fold change of downregulated genes (CDC25C, PLK1, EZH2, BIRC5) in stable SK-Hep–1 cell line transfection with overexpression vector pcDNA3.0-MEG3 compared with stable SK-Hep–1 cell line transfection with blank vector pcDNA3.0 and SK-Hep–1;**Table A-** Clinical Characteristics of the Patients; **Table B-** Primers used for qRT-PCR.
(DOCX)
