# Supplementary material for: Long Noncoding RNA MEG3 Interacts with p53 Protein and Regulates Partial p53 Target Genes in Hepatoma Cells
Source: PLoS One. 2015 Oct 7;10(10):e0139790. doi: 10.1371/journal.pone.0139790 (PMC4596861; doi:10.1371/journal.pone.0139790)
Supplement: S1 File — Expression of p53 target genes was examined by qRT-PCR and normalized to GAPDH. The bars represent the relative fold change of downregulated genes (CDC25C, PLK1, EZH2, BIRC5) in stable SK-Hep–1 cell line transfection with overexpression vector pcDNA3.0-MEG3 compared with stable SK-Hep–1 cell line transfection with blank vector pcDNA3.0 and SK-Hep–1;Table A- Clinical Characteristics of the Patients; Table B- Primers used for qRT-PCR. (DOCX) [file pone.0139790.s001.docx]

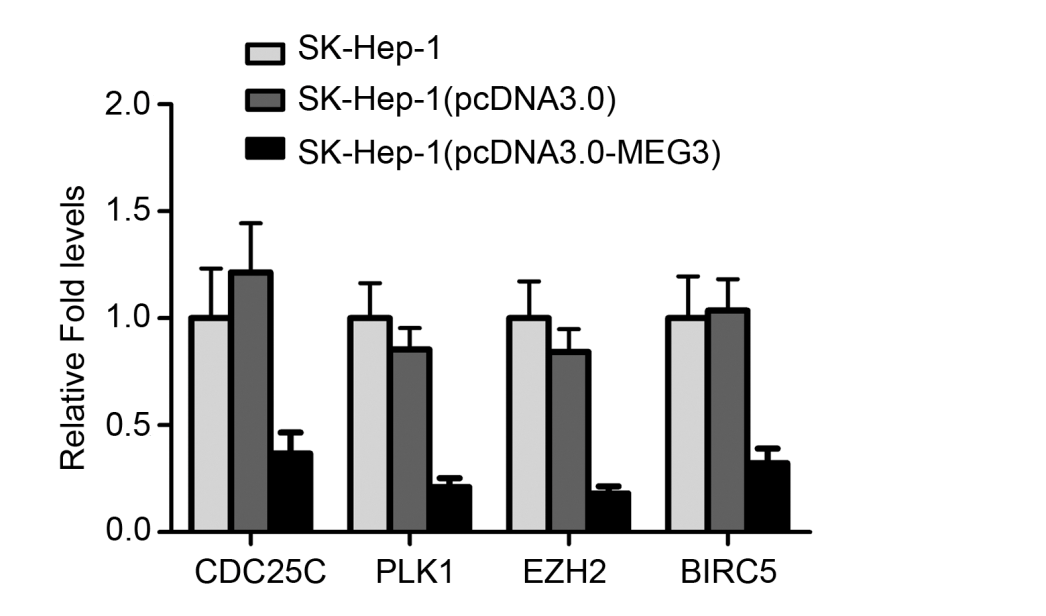


**Figure A.** Deregulated p53 target genes in stable SK-Hep-1 cell line overexpressed MEG3. Expression of p53 target genes was examined by qRT-PCR and normalized to GAPDH. The bars represent the relative fold change of downregulated genes (CDC25C, PLK1, EZH2, BIRC5) in stable SK-Hep-1 cell line transfection with overexpression vector pcDNA3.0-MEG3 compared with stable SK-Hep-1 cell line transfection with blank vector pcDNA3.0 and SK-Hep-1.

| **Table A. Clinical Characteristics of the Patients**. | | | | | | |
| --- | --- | --- | --- | --- | --- | --- |
| **Patient No.** | **Age** | **Gender** | **Tumer Size (cm*cm*cm)** | **HBsAg** | **HCV-Ab** | **Cirrhosis** |
| 1 | 48 | M | 2*2*1.5 | Positive | Negative | No |
| 2 | 40 | M | 2.5*2.5*2 | Positive | Negative | No |
| 3 | 66 | F | 15*7*5 | Positive | Negative | No |
| 4 | 39 | M | 12*11*6 | Positive | Negative | No |
| 5 | 51 | M | 2.5*2*2 | Positive | Negative | Yes |
| 6 | 47 | M | 5*5*4 | Positive | Negative | Yes |
| 7 | 37 | M | 16*4*8 | Positive | Negative | Yes |
| 8 | 52 | M | 8*7*4.5 | Positive | Negative | No |
| 9 | 64 | M | 8*5*4 | Negative | Positive | No |
| 10 | 50 | F | 6*5.5*2.5 | Negative | Negative | No |
| 11 | 52 | M | 7*7*7 | Positive | Negative | Yes |
| 12 | 56 | M | 8*6.5*5 | Positive | Negative | Yes |
| 13 | 48 | M | 2*2*1.5 | Positive | Negative | No |
| 14 | 59 | M | 8.8*8.5*8 | Negative | Negative | No |
| 15 | 41 | F | 12*10*8 | Positive | Negative | No |
| 16 | 34 | M | 10*7*7 | Positive | Negative | Yes |
| 17 | 56 | M | 8*6.5*5 | Positive | Negative | Yes |
| 18 | 37 | M | 16*4*8 | Positive | Negative | Yes |
| 19 | 50 | M | 2.5*1.8*1.5 | Negative | Negative | No |
| 20 | 52 | M | 10.5*8*5 | Positive | Negative | Yes |
| 21 | 42 | M | 13*9*7 | Positive | Negative | Yes |
| 22 | 63 | M | 14*10*10 | Positive | Negative | Yes |
| 23 | 43 | M | 3.5*3*2 | Positive | Negative | Yes |
| HBsAg indicates hepatitis B surface antigen; HCV-Ab, hepatitis C virus antibody; M, male; F, female. | | | | | | |

| **Table B. Primers used for qRT-PCR** | | |
| --- | --- | --- |
| **Gene Symbol** | **Forward** | **Reverse** |
| **BIRC5** | ACCTGAAAGCTTCCTCGACA | AACCCTTCCCAGACTCCACT |
| **CDC25C** | AACTTGGTGGACAGTGAAAT | GCTTCTTGATCTTTCAGGGA |
| **EGR1** | AGGCCGAGATGCAGCTGATGTC | CATCTCCTCCAGCTTAGGGTAGTTG |
| **EZH2** | TTCATGCAACACCCAACACT | GAGAGCAGCAGCAAACTCCT |
| **GADD45A** | CACATTCATCTCAATGGAAGG | CAGGGAGATTAATCACTGGA |
| **PLK1** | TGACTCAACACGCCTCATCC | GCTCGCTCATGTAATTGCGG |
| **SESN2** | TATATCCACTGCGTCTTTGG | AGAGGTTGTACATTCTTCGG |
| **TGFA** | GTCCCCGCTGAGTGCAGA | ACGTACCCAGAATGGCAGAC |
| **MEG3** | GCTCTACTCCGTGGAAGCAC | CAAACCAGGAAGGAGACGAG |
| **GAPDH** | TCAGTGGTGGACCTGACCTG | TGCTGTAGCCAAATTCGTTG |
